# Amyloid plaques beyond Aβ: a survey of the diverse modulators of amyloid aggregation

**DOI:** 10.1007/s12551-017-0271-9

**Published:** 2017-06-19

**Authors:** Katie L. Stewart, Sheena E. Radford

**Affiliations:** Astbury Centre for Structural Molecular Biology, School of Molecular and Cellular Biology, University of Leeds, Leeds, LS2 9JT UK

**Keywords:** Alzheimer’s disease, Amyloid plaques, A-beta, Protein aggregation

## Abstract

Aggregation of the amyloid-β (Aβ) peptide is strongly correlated with Alzheimer’s disease (AD). Recent research has improved our understanding of the kinetics of amyloid fibril assembly and revealed new details regarding different stages in plaque formation. Presently, interest is turning toward studying this process in a holistic context, focusing on cellular components which interact with the Aβ peptide at various junctures during aggregation, from monomer to cross-β amyloid fibrils. However, even in isolation, a multitude of factors including protein purity, pH, salt content, and agitation affect Aβ fibril formation and deposition, often producing complicated and conflicting results. The failure of numerous inhibitors in clinical trials for AD suggests that a detailed examination of the complex interactions that occur during plaque formation, including binding of carbohydrates, lipids, nucleic acids, and metal ions, is important for understanding the diversity of manifestations of the disease. Unraveling how a variety of key macromolecular modulators interact with the Aβ peptide and change its aggregation properties may provide opportunities for developing therapies. Since no protein acts in isolation, the interplay of these diverse molecules may differentiate disease onset, progression, and severity, and thus are worth careful consideration.

## Introduction: what’s in a plaque?

Amyloid plaques, first identified over 100 years ago (Alzheimer 1911), have become an indicative sign of protein misfolding diseases, of which 50 are now identified (Sipe et al. 2016). As the population of the developed world ages, amyloid pathologies are becoming an increasingly grave problem. In 2016, a reported 5.4 million Americans were living with Alzheimer’s disease (AD), perhaps the most well-known amyloid disease, with this number predicted to rise to 13.8 million by 2050 (Assoc. 2016). Thus, understanding the molecular basis of amyloid diseases is of critical importance and has recently been named one of the grand challenges of protein folding, misfolding, and degradation (Goloubinoff 2014).

**Fig. 1:**
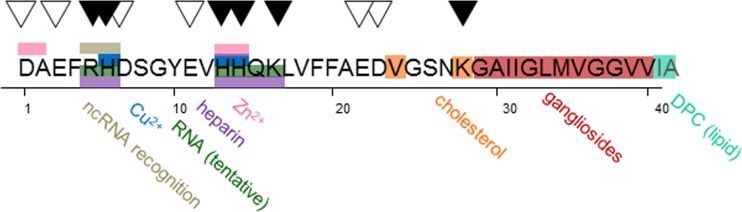
The Aβ42 peptide and its interaction with various plaque components. The sequence of the Aβ peptide, with charged residues (positive *black triangle*, negative *white triangle*). Proposed binding sites of various species discussed in this review are colored by component, using the same coloring for component name below the sequence

**Fig. 2:**
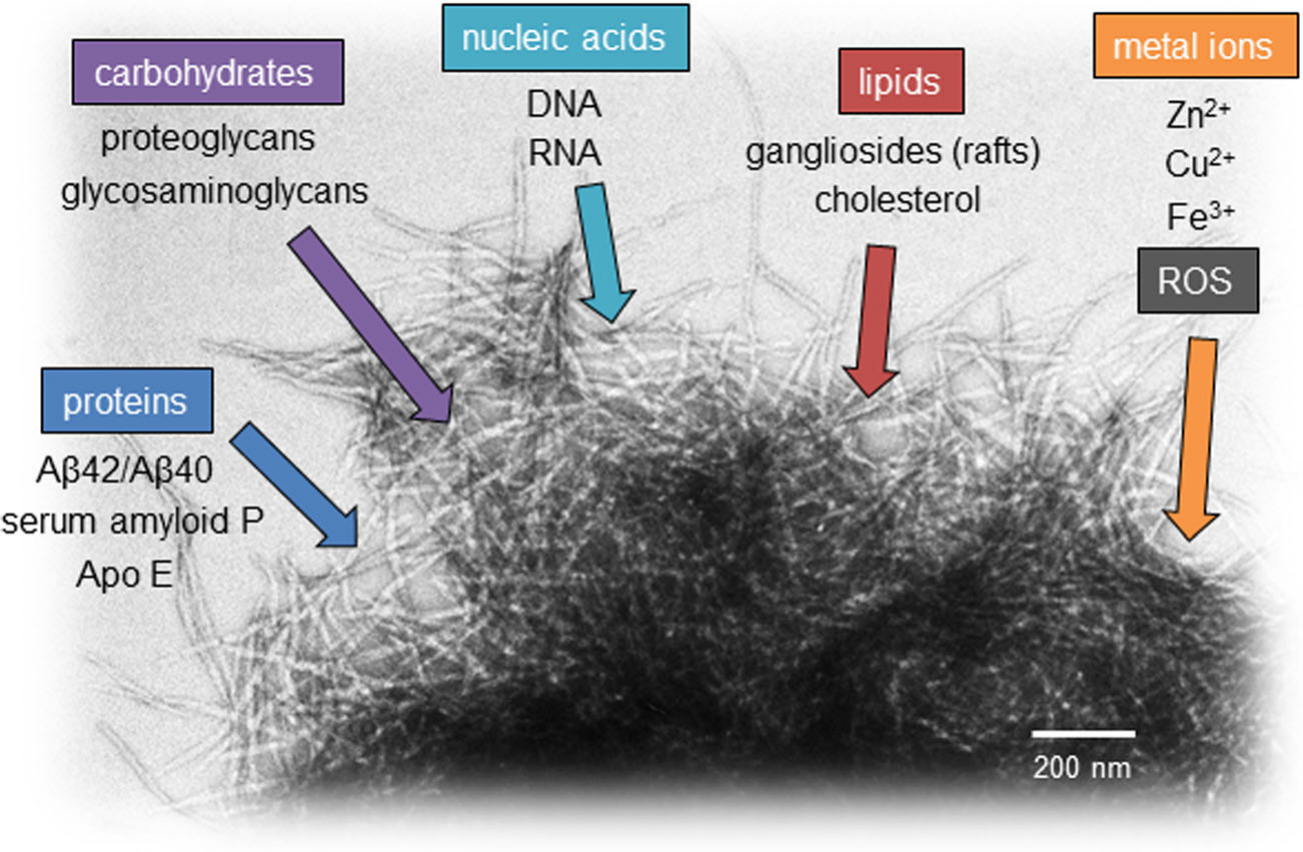
Aβ amyloid plaque contents. Major categories of amyloid plaque components are listed, with particular species shown below. Proteinaceous species discussed in this review are listed, but others are also found within Aβ plaques, see (Liao et al. 2004). The TEM image is comprised of aggregated Aβ42 fibrils collected on a JEOL JEM-1400 microscope, with the *scale bar* indicated on the image

Alzheimer’s disease is postulated to be caused by the formation of senile plaques from the Aβ protein, a soluble, unstructured peptide cleaved from the membrane-embedded amyloid precursor protein (APP) by β and γ secretase enzymes to a length of 38–43 amino acid residues (Knowles et al. 2014). The most well-studied forms of Aβ are the abundant 40-residue form and the highly aggregation-prone 42-residue form. The ratio of Aβ42/40 in the cerebral spinal fluid (CSF) is used as a clinical biomarker to differentiate diagnosis of AD from other forms of dementia (Wiltfang et al. 2007). The Aβ peptide is comprised of a charged N-terminal region (residues 1–22) and hydrophobic C-terminal segment (residues 23–40/42; Fig. 1). The highly hydrophobic central region, residues 16–21 (KLVFFA), is the most aggregation-prone portion of the sequence, and is alone sufficient to cause formation of insoluble fibrils (Gorevic et al. 1987; Preston et al. 2012). Aggregation of both Aβ40 and Aβ42 occur in a nucleation-dependent manner (Meisl et al. 2014), in which several copies of the unstructured peptide contact one another, presumably through hydrophobic (Kim and Hecht 2006) and/or electrostatic (Buell et al. 2013) interactions, forming oligomers and eventually an oligomeric nucleus, which is highly dependent on protein concentration and cellular conditions. Oligomers of Aβ42 in particular (which may be transient or long-lived) have been implicated as cytotoxic disease-causative agents in AD (Haass and Selkoe 2007). Following nucleus formation, aggregation proceeds rapidly through higher-order oligomers to insoluble fibrils, which contain a characteristic cross-β structure (Bonar et al. 1969; Geddes et al. 1968). These fibrils then associate, creating dense mats called plaques, which are highly stable thermodynamic sinks comprised of Aβ40, Aβ42, and other cellular components. Amyloid deposits in the AD brain include intracellular neurofibrillary tangles, principally of the protein tau, and extracellular plaques comprised of the Aβ peptide (Selkoe 2002). Both in vitro (Paravastu et al. 2008) and in vivo (Lu et al. 2013) characterization of Aβ amyloid fibrils have revealed that they are heterogeneous in nature (Eichner and Radford 2011; Tycko 2015), with different fibril morphologies potentially responsible for differences in disease progression between individuals. Plaques are also stockpiles of a wide variety of macromolecular components (Fig. 2), which interact with amyloid fibrils in a variety of ways—both known and unknown—throughout the aggregation cascade (Alexandrescu 2005), and these non-proteinaceous components of amyloid may have important physiological ramifications.

**Fig. 3:**
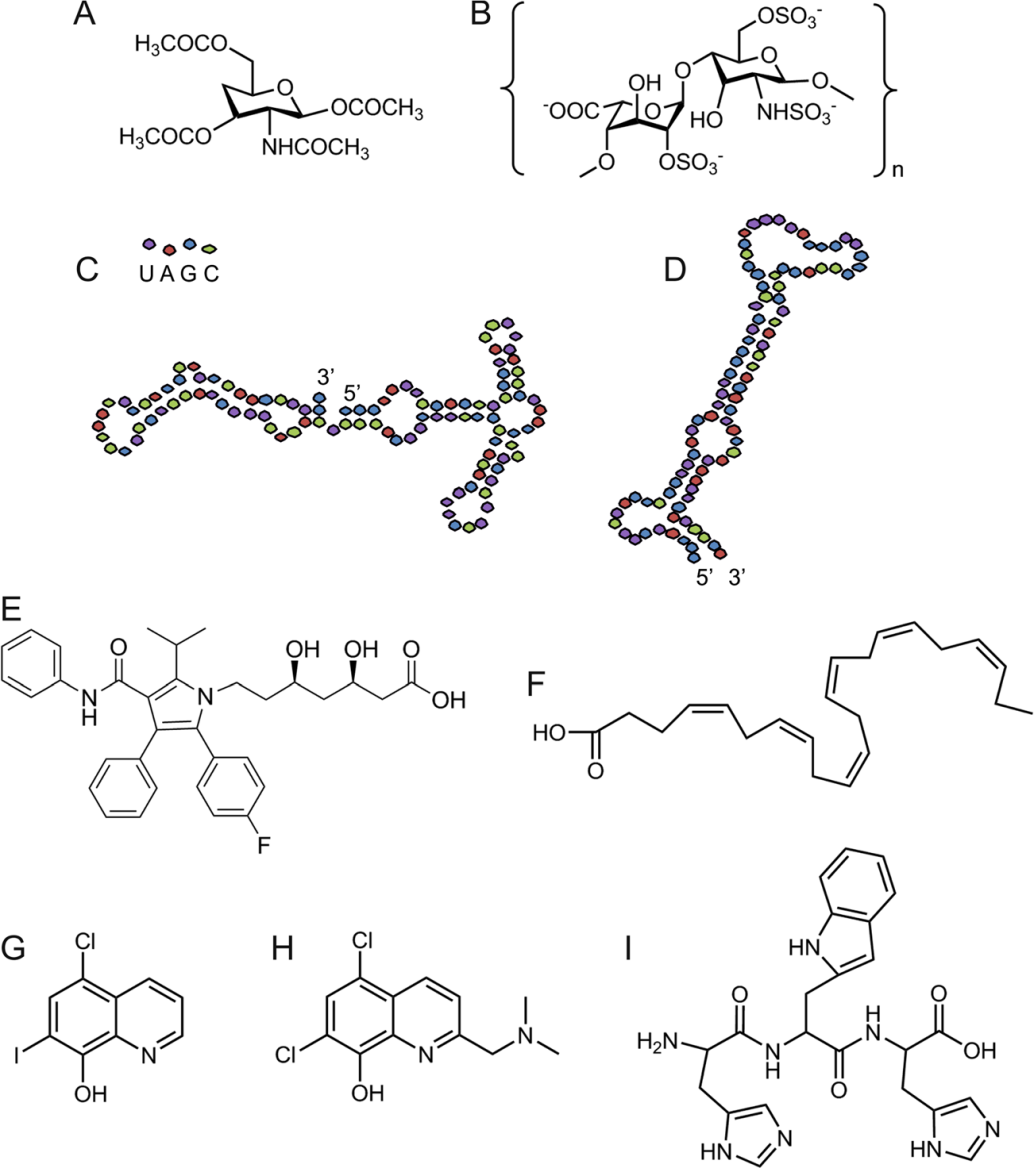
Proposed molecules targeting Aβ aggregation: **a** heparin-based N-acetyl-glucosamine monosaccharide (Kisilevsky et al. 2003); **b** Enoxaparin, a low-molecular-weight heparin (Bergamaschini et al. 2004); **c** RNA aptamer β55 (Ylera et al. 2002), with bases colored as shown; **d** RNA aptamer E2 (Rahimi et al. 2009); **e** the statin Atorvastatin (Lipitor); **f** doxcosahexaenoic acid (DHA); **g** clioquinol; **h** PBT2; **i** tripeptide H-W-H (Caballero et al. 2016)

AD can result from mutations in the Aβ peptide, APP, or related enzymes. This manifestation, termed familial Alzheimer’s disease, is rare, and accounts for <3% of cases. More commonly, AD can arise sporadically late in life, which accounts for ~97% of cases (Masters et al. 2015). Both modes of onset result in a similar disease phenotype: progressive impairment of cognition (Mayeux et al. 2011). While plaque burden is not directly correlated with disease severity (Selkoe and Hardy 2016), the Aβ peptide is regarded as a causative agent in AD (Hardy and Higgins 1992). Particularly in sporadic AD, where the initiation factors of the disease are largely unknown, cellular components are strongly suspect as potential contributors to Aβ-mediated aggregation. Recently, a number of drugs targeting the amyloid cascade have been suggested (Aisen et al. 2006; Bergamaschini et al. 2004), drawn from a variety of engineered and natural binding partners (Fig. 3). However, one of the difficulties facing AD therapeutics includes the fact that Aβ may interact with a wide variety of macromolecules which can alter its aggregation properties or toxicity in vivo and may vary between individuals.

This review provides a brief overview of the major types of non-proteinaceous macromolecules which co-localize with Aβ fibrils in amyloid plaques, and details their binding, aggregation, and cross-reactivity to explore how and why these components are found in in senile plaques. Since a major focus of current AD research involves targeting the aggregation pathway, we also discuss therapeutics inspired by these molecules and their effects on Aβ aggregation. It is worth noting that many proteins also co-localize in amyloid plaques, and these have been quantified by proteomic analysis (Liao et al. 2004; Perreau et al. 2010), but will not be discussed in detail here, aside from the proteins ApoE and serum amyloid P, which are associated with lipid and carbohydrate aggregation factors, respectively (Fig. 2). By focusing on plaques, we assess the variable and complex forces exerted on aggregation of the Aβ peptide in a cellular context, toward therapeutic intervention in AD and other amyloid diseases, and provide some recommendations for future directions.

## Part 1: Carbohydrates

### Proteoglycans and glycosaminoglycans

The term ‘amyloid’, first employed by Rudolf Virchow (Virchow and Chance 1860), means ‘starch-like’, based on an analysis of the first plaques for molecules that were anticipated to be the principal components: starch and cellulose (Sipe and Cohen 2000). It was determined later that the carbohydrate material in plaques consisted of sulfated proteoglycans (Bitter and Muir 1966), an integral part of basement membranes (BM), extracellular surfaces which separate cells and tissue throughout the body. Proteoglycans in the BM form a dense mesh-like network which provides structural support and cellular communication (Varki and Sharon 2009). Experiments utilizing gold-conjugated lectins and fluorescence microscopy have identified that saccharides are found in the periphery of human brain tissue AD plaques (Roher et al. 1993; Szumanska et al. 1987). In particular, the proteoglycan perlecan, which contains 1–3 linear heparan sulfate (HS) glycosaminoglycan (GAG) chains linked to the core protein (Esko et al. 2009), has been shown to bind directly to fibrillar Aβ40 and Aβ42. Other proteoglycans have also been detected in AD amyloid plaques, including the extracellular matrix proteoglycans collagen XVIII and agrin, and the cell surface proteoglycans syndecan 1–3 and glypican 1 (van Horssen et al. 2003). A detailed analysis of the perlecan-binding interface indicated that the GAG HS chains, particularly the negatively charged sulfate moieties, were critical to the interaction (Kisilevsky and Snow 1988; Snow et al. 1987). Several other GAGs which contain sulfate groups have also been detected in AD plaques, including dermatan sulfate (Snow et al. 1992) and chondroitin sulfate (Dewitt et al. 1993; Oohira et al. 2000). Much of the work on the interaction between GAGs and amyloid proteins has subsequently been performed with heparin, a highly sulfated analog of HS which can be produced synthetically (Diaz-Nido et al. 2002; Meneghetti et al. 2015). Heparin binds to Aβ40 similarly to HS and was shown by Castillo and co-workers to contain a high degree of the core sulfate-binding motif present in HS (Castillo et al. 1999).

A wide variety of amyloid proteins bind GAGs, including tau (Goedert et al. 1996), Aβ40/42 (McLaurin et al. 1999a, b), amylin (islet amyloid polypeptide, IAPP) (Jha et al. 2011; Meng and Raleigh 2011), β_2_-microglobulin (Borysik et al. 2007; So et al. 2017), transthyretin (Bourgault et al. 2011), serum amyloid A (SAA) (Ancsin and Kisilevsky 1999), α-synuclein (Madine et al. 2009), and prion (Vieira et al. 2014; Warner et al. 2002). Due to this apparent binding ubiquity, it has been suggested that the interaction between heparin and amyloid is electrostatically-driven, which is supported by the fact that removal of all sulfate groups from heparin impairs its binding to Aβ40 (Castillo et al. 1999). An investigation of interaction sites on all known heparin-binding proteins (Cardin and Weintraub 1989; Sobel et al. 1992) yielded several generalized heparin-binding motifs: XBBXBX, XBBBXXBX, and XBBBXXBBBXXBBX, where B is a basic residue and X is any other residue. The fragments of sequence-separating basic residues suggest a possible role for protein structure in heparin binding, allowing multiple basic residues to be brought into proximity by protein folding. In support of this hypothesis, heparin has been shown to bind with differing affinity to a variety of Aβ40 fibril morphologies comprised of an identical sequence (Madine et al. 2012; Stewart et al. 2016), indicating that GAG binding, despite its apparent ubiquity, can also exhibit specificity. Additionally, individual residues on a given amyloid chain have been shown to alter heparin binding in SAA (Ancsin and Kisilevsky 1999) and Aβ1–28 (McLaurin and Fraser 2000), indicating that binding is not generic across different basic residues. An investigation of the role of sulfate groups on binding to a specific morphology of Aβ40 fibrils indicates that the geometry of the GAG molecule is also important for binding to amyloid fibrils (Lindahl et al. 1999) (Stewart et al., unpublished). Thus, the heparin–amyloid interaction is governed both by general electrostatic complementarity and more specific topological requirements for both the protein and GAG chain.

Considering the Aβ peptide specifically, GAGs have been shown to reduce cellular toxicity in Aβ25–35 and Aβ42 (Bravo et al. 2008; Woods et al. 1995), to stabilize fibrils against degradation in Aβ42 (Valle-Delgado et al. 2010), and to accelerate fibril formation in Aβ40 and Aβ42 (Castillo et al. 1999). GAGs have also been proposed to perform a templating role in amyloid aggregation, providing a scaffold for subunits to self-associate (Motamedi-Shad et al. 2009a; Solomon et al. 2011), and to attenuate cellular toxicity by favoring a benign, alternate aggregation pathway (Bravo et al. 2008; Motamedi-Shad et al. 2009b). GAG molecules are also intimately tied to AD plaque formation and amyloid burden. Recent work by Liu and co-workers removed a critical component of HS biosynthesis, the gene *Ext1*, creating a line of HS-deficient mice (Liu et al. 2016). In these animals, soluble Aβ clearance was increased and amyloid plaque deposition was reduced (Liu et al. 2016). *Ext1* inactivation also reduced neuroinflammation as measured by a reduction in TNF-α and IL-6 inflammatory cytokines, in keeping with heparin’s traditional medicinal use as an anticoagulant (Bjork and Lindahl 1982). A related study overexpressing heparinase, the enzyme which degrades heparin and heparan sulfate, also reduced plaque burden (Jendresen et al. 2015). These studies indicate that GAGs are important for Aβ deposition in amyloid plaques. However, whether this outcome exacerbates or retards disease progression remains unclear.

### Serum amyloid P: a lectin-binding protein

In addition to proteoglycans, Aβ amyloid plaques also contain carbohydrate-binding proteins whose levels are altered in AD. One of the most well-characterized of these components, found almost universally in amyloid plaques, is the Ca^2+^-dependent protein of the innate immune system, serum amyloid P (SAP) (Pepys et al. 1994). This five subunit pentraxin interacts with GAGs during its normal cellular function and is able to neutralize their anticoagulant activity (Williams et al. 1992). Additionally, SAP binds to a variety of amyloid proteins, including Aβ fibrils isolated from AD plaques, and stabilizes them from degradation (Tennent et al. 1995). Based on refolding studies using lactate dehydrogenase, SAP has been suggested to perform a chaperone-like role in reducing aggregation generally (Coker et al. 2000). Recent findings point to Ca^2+^-dependent binding of the SAP pentamer to Aβ40 in both monomeric and fibril forms (Ozawa et al. 2016), although the precise molecular details of these interactions are not known. Since SAP has the ability to bind both GAGs and amyloid fibrils, it is likely an important modulator of protein aggregation and plaque formation.

### Short glycosaminoglycans as amyloid therapeutics

As noted above, heparin has historically been administered as an anticoagulant (Bjork and Lindahl 1982), a property which is increasingly recognized as important for AD (Akiyama et al. 2000; Heppner et al. 2015). As GAGs are small, natural biomolecules, they are able to cross the blood–brain barrier, alter Aβ aggregation, and mitigate cytotoxicity (Bergamaschini et al. 2009). Kisilevsky and co-workers have screened an array of different short GAGs comprised of one to three disaccharide units with the hope of outcompeting full-length GAGs and other negatively charged molecules for amyloidogenic monomeric peptides (Fraser et al. 2001; Kisilevsky and Szarek 2002; Kisilevsky et al. 2003). These authors have identified several GAG mimetics which inhibit SAA amyloid aggregation in a transgenic mouse model (Kisilevsky et al. 2003); one such molecule, a derivative of N-acetyl-glucosamine, is shown in Fig. 3a, and could logically be utilized additionally in targeting Aβ aggregation. Relatedly, Enoxaparin, a low-molecular;weight heparin, acting by a similar mechanism to the short GAGs, was shown to reduce plaque accumulation in an AD mouse model, while also reducing cytotoxicity and inflammation (Bergamaschini et al. 2004) (Fig. 3b). In a randomized pilot study, Enoxaparin was shown to increase the concentration of Aβ42 in cerebrospinal fluid. A recent study, however, has called into question the benefit of increased soluble Aβ in the treatment of AD (Cui et al. 2017), and future work will be needed to resolve the role of GAGs in altering AD symptoms.

## Part 2: Nucleic acids

DNA was initially recognized as a molecule which affects protein aggregation by its ability to promote prion unfolding and conversion into an infective form (Nandi et al. 2002). More recently, nucleic acids have been shown to promote tau aggregation through template-assisted growth (Dinkel et al. 2015) and to bind aggregated Aβ40 (Camero et al. 2013). Nucleic acids also co-localize in amyloid plaques (Ginsberg et al. 1997), and, in particular, neuronal mRNA transcripts have been detected at high levels in these structures (Ginsberg et al. 1999). The binding affinity of RNA molecules to Aβ40 is in the low micromolar range (Rahimi et al. 2009), similar to the affinity for GAGs (Stewart et al. 2016), suggesting that the two molecules may compete for Aβ binding in vivo.

Recently, a systematic study of polyphosphate, the molecular precursor of the nucleic acid backbone, was shown to act as a universal accelerator of amyloid aggregation (Cremers et al. 2016). Using both intracellular and extracellular amyloid proteins, including Aβ42, in both in vitro and in vivo contexts, polyphosphate was shown to be able to accelerate amyloid fibril formation and alter toxicity, stability, and fibril morphology. This work and previous studies (Calamai et al. 2006) postulate that the repeating negatively charged segments of which nucleic acids are comprised act as a β-sheet-stabilizing scaffold for fibril formation, similarly to the role suggested for glycosaminoglycans. The nucleic acid/polyphosphate binding interface for the Aβ peptide, therefore, is most likely located in the same region as the putative GAG binding site, involving positively charged N-terminal residues (Fig. 1). However, whether nucleic acids are able to bind amyloid fibrils universally, or whether binding is more specific to the amyloid and/or nucleic acid structure, as shown for GAGs, remains unanswered.

Nucleic acids may play a larger role in aggregation than simply stabilizing Aβ fibrils in plaques, and have also been observed to affect the structural state of many cellular proteins under stress conditions. Audas and colleagues recently demonstrated that Aβ fibril formation can be a reversed in vivo, via recruitment of long noncoding RNAs (ncRNA), which fine-tune protein expression (Audas and Lee 2016). The authors identified over 180 different types of proteins, including Aβ, which localize in novel cellular compartments they label as ‘A-bodies’ in response to stress (Audas et al. 2016). These proteins contained a similar arginine-histidine sequence targeted by the ncRNAs, which is also found in the N-terminal region of the Aβ peptide (Fig. 1). These surprising findings suggest that ncRNA signals may be lost or compromised in aging, resulting in a prolonged duration of the aggregated stage. Thus, DNA and RNA appear to alter Aβ aggregation processes, as well as being found in plaques. Understanding this interaction more completely, both independently and in combination with possible competing factors such as GAGs, will be key to utilizing both sets of molecules to modulate AD.

### RNA aptamers as amyloid therapeutics

RNA aptamers are short (<100 bp) segments of selection-enriched nucleic acid sequences which are able to bind tightly and specifically to amyloid proteins (Ellington and Szostak 1990; Robertson and Joyce 1990; Tuerk and Gold 1990), and thus can be used to target particular fibril epitopes or stages of disease progression. RNA aptamers are small relative to antibodies and lack the cross-reactivity that antibodies possess (Jayasena 1999). To date, RNA aptamers have been developed which limit prion infectivity (Proske et al. 2002; Rhie et al. 2003), change aggregation co-assembly mechanisms (Sarell et al. 2014), and target specific amyloidogenic proteins (Bunka et al. 2007). Aptamers have also been utilized to select for Aβ-binding partners which disrupt amyloid aggregation. For example, Ylera and colleagues developed RNA aptamers which bind Aβ40 fibrils with nanomolar affinity, which could potentially be utilized as therapeutic or diagnostic tools (Ylera et al. 2002) (Fig. 3c). Relatedly, RNA aptamers developed against Aβ40 fibrils were able to recognize these structures even when thioflavin T, a common amyloid fibril identifier, could not (Rahimi et al. 2009) (Fig. 3d). Aptamers thus provide a hopeful approach to identifying or targeting amyloid proteins. To date, however, despite the potentials of RNA aptamers, these molecules have not yet been shown to provide clinical benefit.

## Part 3: Lipids

A recent assessment of lipid content of AD plaques in human brain tissue revealed that lipids co-localize with cross-β fibrils in amyloid plaques and differ in their organization and composition in the plaque core versus periphery (Kiskis et al. 2015). Lipid structures may therefore potentially trap early-stage amyloidogenic proteins, increasing their local concentration and promoting aggregation. Membranes may also induce pre-fibril forms of amyloid to form pore structures, leading to dysregulation of metal ions and other small molecules, and resulting in a host of downstream consequences for cell homeostasis.

### Lipid rafts and gangliosides

Although a number of lipid surfaces have been shown to affect amyloid aggregation, lipid rafts have emerged as a key binding interface for Aβ40 and Aβ42 (Kim et al. 2006; Wong et al. 2009). Lipid rafts are heterogeneous collections of dynamic gangliosides, sphingolipids, and cholesterol molecules which laterally associate and are detergent-resistant (Simons and Ikonen 1997). These membranes are involved in cellular import/export and signal transduction, including neurotransmission (Colin et al. 2016). The ganglioside and cholesterol composition of lipid rafts has been shown to affect the oligomerization of Aβ42 (Kim et al. 2006), while ganglioside-enriched brain lipid rafts have been shown to accelerate Aβ40 fibril assembly, alter fibril morphology, and increase neurotoxicity (Matsuzaki et al. 2010; Okada et al. 2008). During binding, the soluble Aβ peptide is converted into a helical fold (Fletcher and Keire 1997; Shao et al. 1999) which, upon reaching a critical concentration, is then able to convert to a β-sheet conformation (Matsuzaki 2007). Similar aggregation pathways have been observed in IAPP (Wakabayashi and Matsuzaki 2009) and α-synuclein (Di Scala et al. 2016; Rao et al. 2010), suggesting a generic scaffold-like interface for multiple amyloid proteins. Using the dye diethylaminocoumarin, Ikeda and Matsuzaki showed that binding of Aβ40 to gangliosides involves both hydrophobic and hydrogen-bonding interactions (Ikeda and Matsuzaki 2008), by contrast with the electrostatic interactions which dominate RNA binding and are also involved in GAG binding. The authors of this study map the interaction using an Aβ29-40 fragment, which localizes the ganglioside-binding interface specifically to the C-terminal hydrophobic region of the full-length protein (Fig. 1).

Unlike other effectors of amyloid aggregation, membranes may not only induce cross-β aggregates, but may also facilitate novel amyloid structures, including pores (Arispe et al. 1993). Indeed, pore-like structures comprised of protofibrils have been observed in postmortem AD patients (Inoue 2008). Pore formation is particularly dangerous as it causes increased cellular toxicity, increased passive transport of small molecules, and ultimately cell death (Butterfield and Lashuel 2010). Aβ42 is slightly more hydrophobic than Aβ40, due to its extended C-terminus, and, since hydrophobicity is an important property for membrane interactions, differences between the two peptide forms have been assessed. Sera-Batiste and co-workers systematically monitored the aggregation properties of Aβ40 and Aβ42 in the presence of membranes of various composition over time using gel filtration. The authors observed that Aβ42 reconstituted in dodecylphosphocholine micelles produced homogenous oligomers which were able to form β-barrel pore structures, while Aβ40 reconstituted under the same conditions formed fibrils which lacked pore-like properties (Serra-Batiste et al. 2016). Computational modeling of Aβ42-lipid pores proposed that these structures could be composed of several hexameric units, which associate into a stable 36-stranded β-barrel with a diameter large enough to accommodate metal ions (Shafrir et al. 2010). These results suggest differences in the hydrophobicity of Aβ peptide sequences lead to differences in their behavior with membranes, which may reflect the more toxic nature of Aβ42 compared with Aβ40. Membranes, in particular gangliosides, may play a critical role in Aβ fibril assembly and toxicity. Their co-localization in Aβ plaques suggests that the composition and properties of lipids cannot be ignored as a contributing factor to AD.

Lipids may also be intimately involved with reactive oxygen species (ROS) generation, particularly as a source of oxygen radicals. ROS damage has been linked to membrane binding by both Aβ42 oligomers and fibrils in cell culture (Cenini et al. 2010), and may also occur by dysregulation of metal ions, potentially as a result of lipid-mediated Aβ42 pore formation (Perry et al. 2002). Additional implications of ROS will be discussed in “Part 4”.

### Cholesterol and apolipoprotein E

Another key component of lipid rafts is cholesterol, a molecule which has garnered significant attention for its role in heart disease. High cholesterol diets have also been implicated in causing AD-like behavioral and pathological symptoms in laboratory animals, including increased Aβ42 production (Ullrich et al. 2010). Both cholesterol and apolipoprotein E (discussed below) have been observed in the core of AD plaques (but not diffuse plaques) of transgenic mice, suggesting a direct interaction with Aβ fibrils (Burns et al. 2003). While cholesterol is not required for Aβ oligomerization (Kim et al. 2006), it has been shown to accelerate binding of the Aβ5-16 fragment to gangliosides, by stabilizing an optimal ganglioside dimer conformation (Fantini et al. 2013). Additionally, cholesterol has been shown to bind directly to fragments of the Aβ peptide through C-terminal residues V24 and K28 based on in vitro and in silico measurements (Di Scala et al. 2013), highlighting the importance of both charge and hydrophobicity. A link between cholesterol and copper ions as AD risk factors has been proposed based on patient studies, although their combined role in affecting disease progression has not been fully determined (Morris et al. 2006).

Apolipoproteins are involved in cholesterol transport through the nervous system by binding to cell surface receptors including proteoglycans. Perhaps the most well-studied apolipoprotein in the context of AD is the E class (ApoE), which has been shown to affect Aβ production, deposition, and clearance in sporadic Alzheimer’s disease and is also found in senile plaques. In APP transgenic mice, knockout of ApoE prevented amyloid deposition; instead, the animals formed only diffuse plaques (Holtzman et al. 2000). Alleles of ApoE, containing different residues at positions 112 and 158 (Ɛ2: C112/C158, Ɛ3: C112/R158, and Ɛ4 R112/R158) regulate the binding preferences for high- (Ɛ2) versus low- (Ɛ4) density lipoproteins (Puglielli et al. 2003), which affects membrane composition. Recently, it was shown that ApoE alleles directly stimulate Aβ production, with Ɛ4 > Ɛ3 > Ɛ2 (Huang et al. 2017). The allelic variation of the isoforms therefore is closely linked to AD, with 40% of individuals with AD expressing the Ɛ4 isoform (Farrer et al. 1997). Direct binding between ApoE and the Aβ peptide has been suggested (Carter 2005; Strittmatter et al. 1993); however, Verghese and colleagues have utilized in vitro and in vivo measurements in cerebrospinal and interstitial fluid analyzed by gel filtration to show minimal binding between ApoE and soluble Aβ40/42 (Verghese et al. 2013). Interestingly, ApoE processing has been linked recently to iron metabolism, indicating a role for this component in the maintenance of brain metal homeostasis, with potential implications for AD, as described in “Part 4” (Belaidi and Bush 2016). Thus, ApoE and cholesterol are closely linked, affecting lipid membrane composition and ultimately Aβ aggregation and toxicity. Research continues into the nuances of this pathway and its implications in cognitive decline.

### Lipids as therapeutics

Statins, which reduce the risk of cardiovascular disease by altering cholesterol levels, have been shown to lower the risk of developing AD (Jick et al. 2000) (the most highly-prescribed statin is shown in Fig. 3e). To date, studies assessing the role of statins on AD have been hampered by generalizations between various statins which vary in blood–brain barrier penetration and thus potentially their effectiveness, as well as differences in dosage and duration between experiments (Shepardson et al. 2011). A longitudinal study measuring rates of decline in cognition in adults with normal cognition and mild cognitive impairment who used statins (with no particular type of statin specified) found reduced cognitive decline over time in adults initially with normal cognition, but no effect on patients exhibiting mild cognitive decline (Steenland et al. 2013), relative to statin non-users. Thus, statins may prove to be a protective factor for AD. However, much more data are required to determine the duration statins must be administered to show a protective effect and whether this effect is universal. The natural product omega-3-fatty acids which contain doxcosahexaenoic acid (DHA) (Fig. 3f) affect lipid raft composition, size, and stability, resulting in changes in membrane permeability and receptor binding (Colin et al. 2016). A recent review highlights that DHA, while not effective in studies comprised of the general population, is a particularly potent therapeutic for carriers of the ApoE ε4 isoform (Yassine et al. 2017). This finding represents one of the first potential treatments for carriers of the most dangerous ApoE allele. DHA can be administered with relatively few side effects, making this an attractive, potentially long-term, strategy for older individuals who do not yet show symptoms of AD.

## Part 4: Metal ions

One prolific area of research on AD is the binding of metal ions to Aβ, inspired by the finding that various metals are found concentrated in senile plaques, relative to other tissues (Faller 2009; Maynard et al. 2005; Tougu et al. 2011). Levels of zinc, iron, and copper ions in the brain, although normally tightly regulated, fluctuate substantially upon neuronal activation, resulting in pools of ions that may not be cleared as readily in aged individuals (Faller 2009). These ions may also play a role in ROS generation, which may occur through metal ion reduction (Huang et al. 1999). Direct binding of Aβ40 to Cu^2+^ and Zn^2+^ has been observed, implicating the peptide as an aberrant metal chelator or indirectly in causing lipid-based pore formation which alters the brain metal ion balance. Other metal ions have also been investigated in connection with AD including Ca^2+^, Mg^2+^, Mn^2+^, and Al^3+^. However, limited studies of these ions to date have pointed to roles as upstream or indirect effectors of amyloid aggregation (Hare et al. 2016; Khachaturian 1987; Li et al. 2013). The latter set of ions will not be addressed further here. Instead, we focus on three known effectors of AD which are found elevated in amyloid plaques: Cu^2+^, Zn^2+^, and Fe^3+^.

### Copper ions

**Fig. 4:**
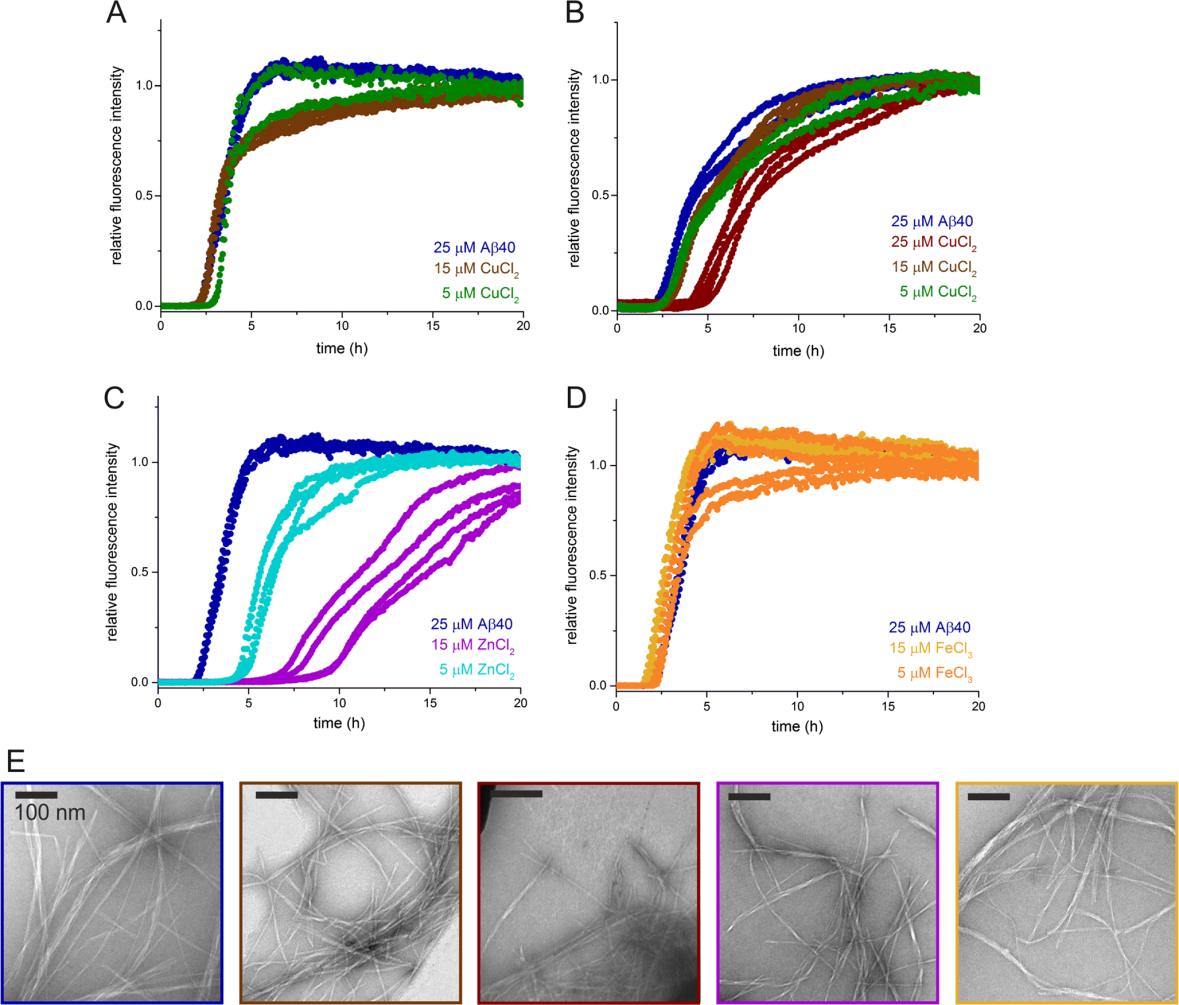
The effects of substoichiometric quantities of various metal ions on the rate of aggregation of monomeric Aβ40. 25 μM monomeric Aβ40 in the presence of **a** CuCl_2_ at pH 7.5, **b** CuCl_2_ at pH 6.5, **c** ZnCl_2_ at pH 7.5, and **d** FeCl_3_ at pH 7.5. All contain 10 μM thioflavin T in 25 mM NaH_2_PO_4_ at 37 °C, and were analyzed quiescently on a Fluorostar Omega plate reader (BMG Labtech) with an excitation wavelength of 440 nm and an emission wavelength of 480 nm. Multiple replicates are shown in the same color. **e** TEM images of fibrils formed after 24 h. *Border color* indicates sample shown; from *left* to *right*: 25 μM Aβ40 (alone) at pH 7.5, 25 μM Aβ40 with 15 μM CuCl_2_ at pH 7.5, 25 μM Aβ40 with 25 μM CuCl_2_ at pH 6.5, 25 μM Aβ40 with 15 μM ZnCl_2_ at pH 7.5, 25 μM Aβ40 with 15 μM FeCl_3_ at pH 7.5

Perhaps the most extensively studied and well-characterized metal ion bound by Aβ is copper. This interaction depends on a number of factors, including the pH of the amyloid environment, concentration of metal ions relative to Aβ, and the oxidation state of the metal. Aβ40-copper binding in both the 2^+^ and 1^+^ oxidation states has been pinpointed principally to the three histidine residues at positions 6, 13, and 14 (Fig. 1). Copper ion binding occurs more readily at mildly acidic pH resulting in characteristic insoluble plaques, while at physiological pH, soluble Aβ40 and Aβ42 aggregates have been observed (Atwood et al. 1998; Mold et al. 2013). In a series of elegant studies using electron paramagnetic resonance, the binding site of Cu^2+^ was mapped principally to H6 and either H13 or H14, with the interaction region alternating on successive fibril strands of Aβ40 (Gunderson et al. 2012). Additional characterization showed that Cu^2+^ does not alter the fibril architecture or aggregation pathway (Karr et al. 2005; Karr et al. 2004), and could bind oligomeric (Karr and Szalai 2008; Sarell et al. 2010) or monomeric (Pedersen et al. 2015) Aβ40. One consequence of this binding arrangement is its ability to induce fibril–fibril association (cross-linking), as observed in aggregation experiments with substoichiometric Cu^2+^ at low pH (Karr and Szalai 2008; Sarell et al. 2010). In our own work, we have observed a Cu^2+^-specific effect on the aggregation rate of Aβ40 under low (pH 6.4), but not neutral (pH 7.4), conditions (Fig. 4a, b), in agreement with the importance of histidine protonation in this interaction. Interestingly, the GAG heparin has also been shown to bind Cu^2+^ ions, causing a change in heparin chain conformation, which may have additional implications for cooperativity or competition with the Aβ peptide (Rudd et al. 2008). The binding site for Cu^1+^ ions has also been characterized in Aβ40 and forms a linear binding arrangement involving H13 and H14 with similar stoichiometry to Cu^2+^ ions (Shearer and Szalai 2008).

The interaction of copper ions with Aβ40 and Aβ42 has also been studied in regard to ROS generation, particularly with oligomeric and fibrillar Aβ species. However, whether Cu^2+^ binding to Aβ species increases or decreases ROS is debated. Mayes and co-workers have suggested that Aβ42 fibrils can degrade peroxide in a Cu^2+^-binding dependent manner, with the highest ROS generation at a 1:1 ratio of Aβ:Cu^2+^ (i.e. saturated binding) (Mayes et al. 2014). In contrast, Pedersen and colleagues demonstrated that ROS generated from oxygen and ascorbate was reduced in the presence of fibril forms of Aβ40 and α-synuclein compared with Cu^2+^ alone (Pedersen et al. 2016). This finding suggests that ROS production is initiated by free metal ions rather than aggregation of the Aβ peptide, and that ROS in AD plaques results from the prevalence of free, rather than bound, metal ions. Regardless of the initiating species, ROS generation is strongly correlated with AD, and oxidative damage is a major factor in disease progression (Huang et al. 2016; Perry et al. 2002).

### Zinc ions

Zn^2+^ ions are also elevated in AD amyloid plaques, and have been shown both to accelerate (Bush et al. 1994) or retard (Abelein et al. 2015; Sarell et al. 2010) Aβ40 aggregation at physiological pH in vitro, depending on the conditions used. Under similar conditions to those used by Abelein, we observed an increase in the lag time of Aβ40 aggregation with increasing concentrations of Zn^2+^ ions (Fig. 4c). Similarly to Cu^2+/1+^, the Zn^2+^ binding site involves residues H13 and H14, and also the N-terminus of the protein, although binding does not appear to be mediated by histidine protonation as was observed for Cu^2+/1+^ (Yang et al. 2000). A detailed characterization of Zn^2+^ binding site by Rezaei-Ghaleh and co-workers by nuclear magnetic resonance (NMR) showed that other regions of the Aβ40 peptide, particularly residues D23–G29 (Fig. 1), may change conformation in response to Zn^2+^ ions, indicating that the binding interaction has global implications for Aβ structure (Rezaei-Ghaleh et al. 2011). Additionally, Zn^2+^ has been shown to promote nucleic acid association with Aβ42, with particular importance for histidine residues 6 and 13 (Khmeleva et al. 2016). Zn^2+^ has also been shown to play a protective role in ROS generation, by competing for Aβ40/42 fibril binding with Cu^2+^ (low μM/high pM for Cu^2+^ vs. low- to mid-μM for Zn^2+^ dissociation constants) (Faller and Hureau 2009). In the presence of both ions, ROS generation was shown to be reduced relative to Cu^2+^ alone (Mayes et al. 2014), suggesting, in agreement with other results (Cuajungco et al. 2000), that Zn^2+^ binding limits ROS generation.

### Iron ions

Brain Fe^3+^ levels have been shown to be elevated in autopsy studies of AD patients (Loef and Walach 2012) and are correlated with oxidative damage (Casadesus et al. 2004), which Fe^3+/2+^, like Cu^2+/1+^, may promote (Wang and Wang 2016). The addition of a 10-fold molar excess of Fe^3+^ has been reported to alter Aβ42 fibril morphology, resulting in shorter, curved fibrils with elevated toxicity (Liu et al. 2011). In a study of the binding of 20-fold excess of various metal ions to the Aβ40 peptide, Clements and co-workers demonstrated that Zn^2+^ and Cu^2+^ binding were stronger than Fe^3+^ and Al^3+^, which were unable to displace Zn^2+^ (Clements et al. 1996). Substoichiometric amounts of Fe^3+^ did not alter the rate of Aβ40 aggregation in our kinetics survey, arguing against a significant role under the conditions tested (Fig. 4d). As mentioned previously, iron levels are directly correlated with the ApoE isoform. These findings indicate that individuals with the ApoE ε4 allele contain elevated levels of the iron storage protein ferritin in the cerebrospinal fluid (Ayton et al. 2015), which cause elevated brain-iron levels in AD. Therefore, while Fe^2+/3+^ ions play a role in amyloid pathology, they may do so indirectly in their role as a redox-active and pathway-associated metal ion, rather than as a direct binding partner of the Aβ peptide.

### Metal ion chelators as amyloid therapeutics

A number of metal ion chelators have been investigated as possible therapeutics, with a focus on altering the soluble cellular pool of metal ions. Iodochlorhydroxyquin (clioquinol) (Fig. 3g), a chelator of copper and zinc ions, was able to reduce plaque burden and memory loss in animal models (Cherny et al. 2001) and in early-stage human clinical trials (Regland et al. 2001). In pilot phase 2 clinical trials, treatment with clioquinol was significant in reducing memory loss in patients with severe dementia, and was shown to reduce plasma Aβ42 levels while increasing plasma Zn^2+^ levels (Ritchie et al. 2003). A related chelator, PBT2 (Fig. 3h), was developed to be more tolerant in higher doses than clioquinol, and has undergone phase II clinical trials. In an initial 12-week study, a 250-mg dose was more effective at preventing cognitive decline than a 50-mg dose (Faux et al. 2010). However, in a longer 52-week trial, PBT2 did not reduce plaque burden or improve cognitive function to a statistically significant extent. Recently, Caballero and co-workers have designed peptide fragments containing one to two histidine residues which showed higher affinity for Cu^2+^ ions than the Aβ40 peptide, and also showed reduced amyloid toxicity and reduced copper-generated ROS (Caballero et al. 2016) (Fig. 3i). While these fragments are now only at a preliminary test phase, they may prove to be useful therapeutic scaffolds for future metal ion chelators. There has also been an increasing focus in patient studies on the role of dietary metal ions in AD. An overview of published clinical trials and cross-section studies (Loef and Walach 2012) concluded that most trials to date have produced inconclusive results, primarily due to the study duration or inability to control for dietary or lifestyle variables. As mentioned previously, a plausible link between copper ions and high cholesterol has emerged, but specific details of the interaction must be elucidated further (Morris et al. 2006). Taken together, these results indicate that altered metal ion chelation and/or consumption, while important for AD pathology, is not alone sufficiently potent to significantly inhibit AD, and must be considered alongside other factors.

## Conclusions: commonalities, competition, and cross-coordination

Plaques are complicated assortments of aggregated protein and other co-effectors of the aggregation process (Fig. 2). The balance of such molecules in the cellular environment, under both healthy and disease conditions, may alter the Aβ aggregation rate and ability to interact with additional extracellular factors. Figure 1 shows the proposed binding sites on Aβ40/42 for a number of the molecules detailed in this review. Although a large number of binding partners may compete for the histidine residues in the N-terminal region of Aβ40/42, there are other binding sites distributed throughout the sequence, suggesting that the Aβ peptide may interact with multiple binding partners, exhibiting various charges or lack thereof, simultaneously or in succession. Additionally, due to differences between the aggregation propensities and intermediate states sampled in Aβ40 versus Aβ42 (Bitan et al. 2003; Meisl et al. 2014), preferences toward binding partners may differ between Aβ forms. This complicated interplay may be responsible for the variation observed in fibril morphology (Annamalai et al. 2016; Tycko 2015) and rate of disease progression, which can fluctuate in sporadic AD from months to decades (Komarova and Thalhauser 2011; Thalhauser and Komarova 2012).

**Fig. 5:**
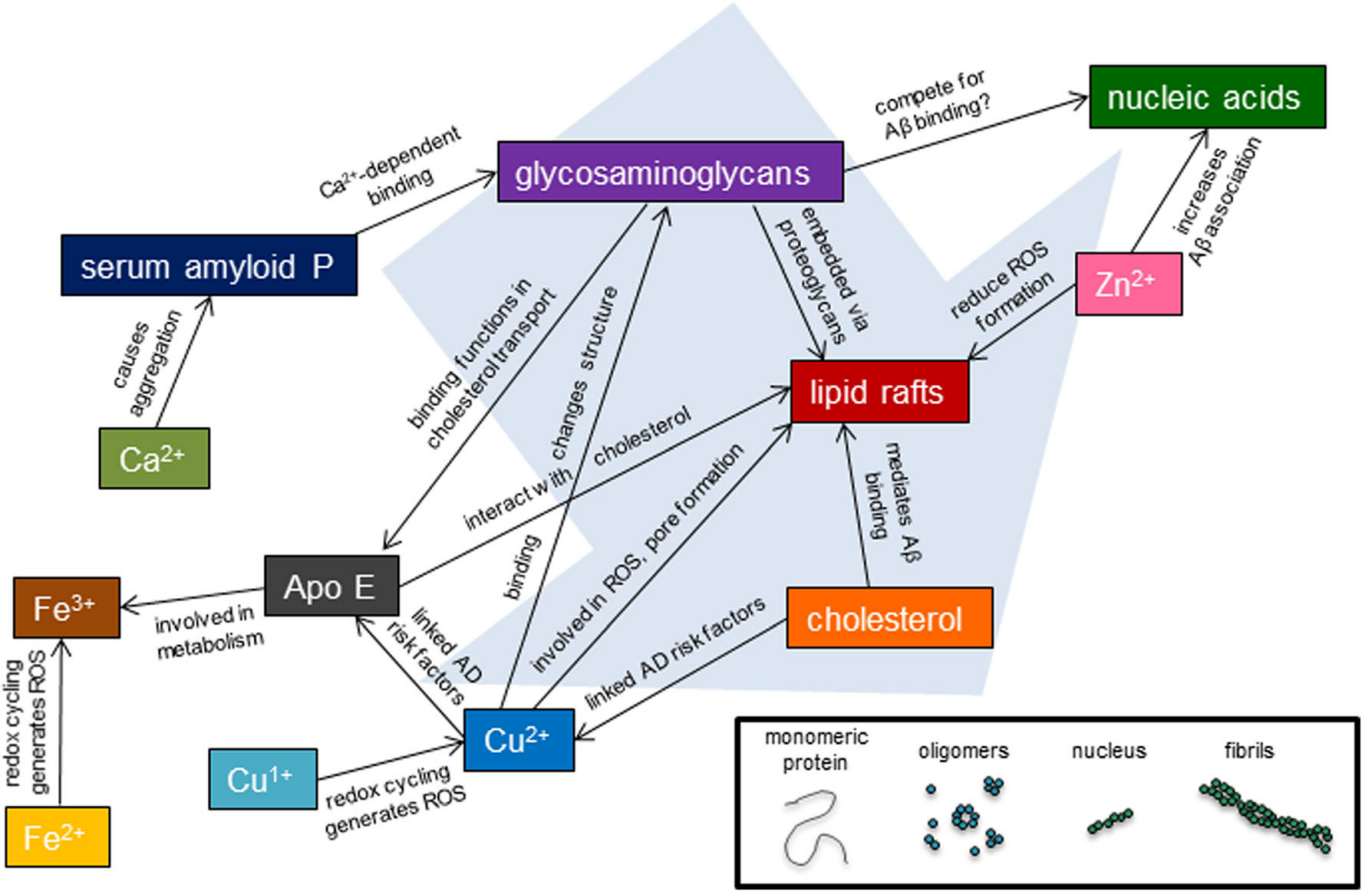
Cross-interactions of plaque components. Plaque components colored as in Fig. 1 (where applicable). *Lines connecting species* describe interactions. Although all these species are found in amyloid plaques (fibrils), their interactions with earlier stage Aβ is also possible. A schematic of the Aβ peptide aggregation pathway is shown at the *bottom right*

To date, no therapeutic has been identified which is able to fully mitigate AD. Perhaps this is because many drugs to date (Fig. 3) have targeted a single extracellular factor, without considering the competition between these molecules, or the fact that such competition may vary greatly between individuals. Future therapeutic strategies must consider the complexity of amyloid aggregation, particularly how the delicate balance of interactions in the brain can not only affect Aβ but how these interactions can also affect one another. One key point of this analysis is how genetic factors, such as ApoE allele, or environmental factors, such as metal ion concentration or cholesterol consumption, alter the production or interaction of the Aβ peptide with other effectors of amyloid aggregation. Figure 5 presents a simplified view of some of these cross-interactions within the complexity of the cellular environment. Clearly, Aβ aggregation is not a simple, linear process. Instead, there is a multitude of factors which mitigate amyloid structure, toxicity, and clearance. Only when these cross-coordination events are considered can the intricacies of the amyloid aggregation cascade be understood and properly targeted.
